# Assessment of Involuntary PFM Contractions in Comparison with Existing Literature and IUGA/ICS Terminology Reports

**DOI:** 10.1007/s00192-024-05729-z

**Published:** 2024-02-08

**Authors:** J. de Jong, F. Burkhard, M. Zwahlen, B. Junginger, C. Dumoulin

**Affiliations:** 1 Graduate School for Health Sciences, University of Bern, Bern, Switzerland; 2 SOMT University of Physiotherapy, Amersfoort, Netherlands; 3 Department of Urology, University Hospital Bern, Bern, Switzerland; 4 Institute of Social and Preventive medicine, University Bern, Amersfoort, Netherlands; 5 Department of Gynecology, Charité University Berlin, Berlin, Germany; 6 Research Centre of the Institut universitaire de gériatrie de Montréal, Montreal, Canada

**Keywords:** Pelvic floor muscle, Involuntary, Reflex, Visual observation, Vaginal palpation, Terminology reports

## Abstract

**Introduction and Hypothesis:**

Involuntary pelvic floor muscle (PFM) contractions are thought to occur during an increase in intra-abdominal pressure (IAP). Although no studies have assessed their presence in women with normal pelvic floor (PF) function, existing literature links the absence of involuntary PFM contractions to various PF dysfunctions. This study rectifies this lacuna by evaluating involuntary PFM contractions during IAP in healthy nulliparous women with no PF dysfunction, using visual observation and vaginal palpation. Results were compared with the literature and the IUGA/ICS Terminology Reports.

**Methods:**

Nulliparous (*n*=149) women performed three sets of three maximal coughs. Visual observation and vaginal palpation were conducted in the standing and supine positions. The women were not instructed to contract their PFMs. Occurrence rates were calculated for each assessment method and position; differences between positions were analyzed using the Chi-squared test.

**Results:**

Rates of occurrence of involuntary PFM contraction were low across both assessments and positions (5–17%). Significant differences were found between standing (5%) and supine (15%) positions for visual observation, but not vaginal palpation (15%, 17% respectively). Occurrence rates also differed compared with the literature and terminology reports.

**Conclusions:**

Contrary to clinical expectations, rates of occurrence of involuntary PFM contraction among our cohort of nulliparous women were extremely low. Digital palpation results showed high agreement with the terminology reports, but only partial agreement was observed for the visual observation results. Our study underscores the need for more research aimed at defining normal involuntary PF functions, a review of our understanding of involuntary PFM contractions, and better standardized guidelines for involuntary PFM assessment methods.

## Introduction

According to the literature, involuntary pelvic floor muscle (PFM) contractions occur without conscious control or effort, and are considered a normal response preceding increased intra-abdominal pressure (IAP), such as during a cough [1–4]. However, the literature lacks consensus in regard to the underlying mechanisms involved in involuntary PFM contractions, and has variously characterized them as a reflex [5, 6], dynamic response [7], co-activation [8–11], pre-programmed activation [12], or feed-forward movement [13]. Moreover, scientific research has focused primarily on assessing and establishing outcome measures for evaluating voluntary PFM contractions, whereas involuntary PFM contractions have received significantly less scientific interest.

The International Urogynaecology Association (IUGA) and the International Continence Society (ICS) have developed standardized Terminology Reports to describe pelvic floor (PF) function and dysfunction [1–4]. These reports are widely used in both clinical practice and research to standardize definitions and output findings for various PF assessment methods. The IUGA/ICS Terminology Reports recommend the use of standard clinical PF assessment methods, such as visual observation and vaginal palpation, to evaluate the PFM response during a cough.

The literature, as summarized in the terminology reports, considers the presence of an involuntary PFM contraction to be normal, and attributes its absence during increased IAP to PF dysfunction [7, 14, 15]. However, most clinical trials have only assessed involuntary PFM contractions in populations with symptoms of PF dysfunction (e.g., urinary incontinence, POP) and, to our knowledge, no studies have evaluated involuntary PFM contractions in healthy women. Consequently, this study was aimed at:

1. Assessing the occurrence rate of involuntary PFM contractions (during a cough) in healthy nulliparous women without PF dysfunction, using visual observation and vaginal palpation in two positions, supine and standing
2. Comparing the assessment results with those of previous studies
3. Comparing the assessment results with commonly used IUGA/ICS terminology reports

## Materials and Methods

### Study Design

This prospective, observational cohort study recruited participants through personal letters sent to patients from two gynecology clinics, as well as through campus advertisements and student mailings at a Swiss university. Inclusion criteria were: female, between 18 and 35 years of age, nulliparous, no current or past history of PFM dysfunction, and the ability to perform a normal or strong voluntary PFM contraction. Participants were excluded if they were currently or previously pregnant, had any form of PFM dysfunction or neurological disorders, were taking medication likely to influence PF function, or had undergone previous PFM training or pelvic surgery. Ethical approval was obtained from the ethics committee of the Bern canton, Switzerland (KEKBE Nr. 2016/12, Amdt. 101/13), and each participant provided written, informed consent before participating in the study.

### Procedure

#### Inclusion Assessments

Potential candidates first completed the validated, self-administered, German pelvic-floor questionnaire (Deutscher Beckenbodenfragenbogen) to exclude those with any form of PF dysfunction [16]. Successful candidates then underwent a vaginal palpation assessment to confirm their ability to perform a voluntary PFM contraction. For this, participants were assessed in a supine position, with a pillow under their head, hips and knees flexed, and their legs slightly parted and supported for optimal PFM relaxation. The examiner (JdJ) inserted a lubricated finger into the candidate’s vagina and palpated the PFMs without overstretching them, while the candidate contracted their PFMs as hard as they could. To be included as a study participant, candidates had to demonstrate a normal or strong voluntary PFM contraction, as measured on the four-point ICS scale (absent, weak, normal, strong) [1].

#### Study Tasks

For both the visual observation and vaginal palpation assessments in the two positions (supine and standing), study participants were instructed to complete a series of tasks beginning with normal breathing for 1 min, followed by three forceful coughs (maximal voluntary coughs) with a 10-s rest between each. The participants first assumed a supine position, as described in the inclusion assessments, and then their habitual standing posture with their feet a shoulder width apart to complete the tasks.

#### Involuntary PFM Contraction Assessments

Visual inspection was performed to assess perineal movement in both the standing and supine positions. To have a minimal effect on the participant’s habitual posture, a mirror was used in the standing position to obtain a better view of the perineum. Per clinical practice, an "inward" movement or "no movement" of the perineum is interpreted as a PFM contraction being “present,” whereas a downward movement indicates the “absence” of a PFM contraction. These interpretations align with the findings from the terminology reports by Messelink et al. and Haylen et al. [1, 2]. All visual observations were performed by the same experienced PF physiotherapist (JdJ) and were supervised by a second PF physiotherapist (BJ) to confirm the involuntary PFM contraction.

Vaginal palpation was performed in the standing and supine positions as explained above, under inclusion assessments [1–4]. Palpation was conducted using one finger, without overstretching the PFMs, to avoid influencing women’s ability to actively contract. Concurrent with the coughs, the examiner identified the presence or absence of an PFM contraction, per the terminology reports [1, 4]. All assessments were carried out by the same examiner (JdJ).

#### Statistical Analysis and Sample Size

Based on pilot data, we expected the proportion of participants with a present PFM contraction, as assessed by visual observation and digital palpation during coughing and forced expiration, to be 70%. Thus, we aimed to recruit 150 participants, to give us a 95% confidence interval with a width of ±7.5%.

Statistical analysis was conducted using SPSS software and Stata 17. Participants’ demographic characteristics (age and BMI) were described using means and standard deviations (SD). We computed the occurrence rate (OR; number/%) of inward or no perineal movements (i.e., the presence of an involuntary PFM contraction) vs downward perineal movements (i.e., absence of an involuntary PFM contraction) as assessed by visual observation, as well as the presence/absence of an involuntary PFM contraction as assessed by vaginal palpation in both the supine and standing positions. Pearson’s Chi-squared test was used to compare occurrence rates between positions (standing, supine) for the visual observation and vaginal palpation assessments. A significance level of *p*<0.05 was considered statistically significant.

## Results

We recruited 172 women, 22 did not meet the inclusion criteria or withdrew, and one did not complete the assessment, leaving 149 participants. The mean age was 26.3 years (SD ± 5.6) and the mean BMI was 21.6 kg/m^2^ (SD ± 4.8). All participants obtained a score of 0/40 on the German pelvic-floor questionnaire (Deutscher Beckenbodenfragenbogen) [16], confirming the absence of PF dysfunction. The evaluating physiotherapist (JdJ) confirmed the presence of a normal or strong voluntary PFM contraction using vaginal palpation.

### Occurrence Rate Findings

#### Visual Observation

For the visual observation in the supine position, the examiner observed an involuntary PFM contraction, as indicated by an inward or no downward movement of the perineum, in 15% of participants (22 out of 149). The absence of a PFM contraction was observed in 85% of participants (127 out of 149). In the standing position, the presence of a PFM contraction was observed in 5% of participants (7 out of 149), and its absence was observed in 95% of participants (142 out of 149).

#### Vaginal Palpation

For the vaginal palpation in the supine position, the examiner evaluated an involuntary PFM contraction to be present in 15% (22 out of 149) and absent in 85% (127 out of 149) of participants. In the standing position, an involuntary PFM contraction was present in 17% (25 out of 149) and absent in 83% (124 out of 149) of participants.

#### Differences between Assessment Positions

A comparison of involuntary PFM contraction results, between the two positions and for each assessment method, indicated a significant difference between the standing and supine positions for the visual observation assessments (*p*=0.003), but no significant differences between the positions for the vaginal palpation assessments (*p*=0.633; Table 1).

**Table 1:** Occurrence rate (OR) of involuntary PFM contractions in the supine and standing positions for the visual observation and vaginal palpation assessments. Differences between supine and standing positions as assessed by visual observation and vaginal palpation were calculated using Pearson’s Chi-squared test

Assessment	*N*	Supine OR, *n* (%)		Standing OR, *n* (%)		Pearson’s Chi-squared	Difference in OR (95% CI)	*p* value
Visual observation	149	22 (15)		7 (5)		8.60	10.1% (3.4 to 16.7%)	0.003
Vaginal palpation	149	22 (15)		25 (17)		0.23	−2.0% (−10.3 to 6.3%)	0.633

## Discussion

### Occurrence Rates

Overall, we observed a markedly low rate of occurrence of involuntary PFM contraction for both visual observation (supine 15%, standing 5%) and vaginal palpation (supine 15%, standing 17%). In the literature, the absence of an involuntary PFM contraction is attributed to PF dysfunction [7, 14, 15]. Consequently, and given that our study exclusively involved healthy, nulliparous women with no PF dysfunction, we expected higher rates of occurrence of involuntary PFM contraction; instead, we found very low rates. Our results raise fundamental questions: what is a normal or abnormal response of the PFMs to IAP? Do the assessment tools accurately measure their intended parameters? These lines of enquiry are pertinent considering the limited published data on evaluating healthy PF function and involuntary PFM contractions using visual observation and vaginal palpation, despite their common usage in clinical practice [17].

### Comparative Analysis with the Literature

We identified two visual observation studies reporting on involuntary PFM contraction occurrence rates during a cough in the literature. The study of Vesting et al. [18], which assessed women (*n*=222) 18 and over, and 3 months postpartum, was comparable with our results. They found an occurrence rate of 9–11%, which aligns with our results of 15%. The study by Devreese et al. [19], which used visual observation to examine perineal response during a cough in various positions (supine, sitting, standing) in continent (*n*=40) and incontinent (*n*=40) women, was not comparable for the following reasons. Participants were assessed at various points (beginning, during, or end) throughout an individual PF exercise program. Consequently, they may have received instructions on how to perform the “Knack,” a learned pre-contraction of the PFMs before coughing, which could have influenced the outcomes. Unlike our study, Devreese et al. defined an involuntary PFM contraction as a coordinated muscle action between the abdominal and PFMs. The continent group demonstrated coordinated PFM contractions and showed high occurrence rates (82.5–97.5%), the incontinent group displayed a lack of coordinated movement, resulting in lower rates (25–35%). In a previous study of healthy women, we evaluated the impact of abdominal movement on the occurrence of an involuntary PFM contraction during a cough, in both supine and standing positions, we found significantly higher occurrence rates of involuntary PFM contractions among women who exhibited an inward abdominal movement compared with those with an outward abdominal movement [20]. Thus, in the Devreese et al. study, both the coordinated muscle action and the potential pre-contraction (Knack) during a cough, could have contributed to this high occurrence rate; hence, it is not comparable with our results [19].

 Six vaginal palpation studies reported involuntary PFM contraction occurrence rates during a cough [18, 19, 21–24]. However, unlike our study, these studies encompassed a wide range of ages and parities, and included varied study populations, both with and without PF dysfunction. These factors likely contributed to the wide variations in reported rates of occurrence of involuntary PFM contraction (from 5.2% to 41.0% in the supine position) [18, 19, 21–24], and make outcome comparisons with our study difficult. Slieker-Ten Hove et al., assessed women from a general population (*n*=41), both with and without PF dysfunction, aged 18–85 (mean age 41), to test the face validity and reliability of the ICS assessment scheme by Messelink et al. [1], and found an occurrence rate of involuntary PFM contraction of 49.5% [21]. In another study (*n*=649 women, age 45–85), Slieker-Ten Hove et al. [23] evaluated the association between the occurrence of an involuntary PFM contraction and muscle strength. Using the ICS scale [1, 4] to assess muscle strength during a cough, they observed a higher rate of occurrence of involuntary PFM contraction in women with weaker PFMs (61.5%) compared with strong PFMs (40.6%). They concluded that PFM strength does not seem to predict the presence or absence of an involuntary PFM contraction [23]. Slieker-Ten Hove et al. also found a lower rate of occurrence of involuntary PFM contractions in younger women (39% in the age group 45–55 years) than in older women (66% in the age group 66–75 years) [23]. The low occurrence of an involuntary PFM contraction among our young, healthy participants seems to echo and possibly provide further support to our findings. Vieira et al. found a higher rate of occurrence of involuntary PFM contractions in continent (43%) versus incontinent (17%) women assessed using vaginal palpation during a cough in a study of 210 women with (*n*=101) and without (*n*=109) urinary incontinence [24]. The authors postulated that the high correlation (82%) was due to a lack of muscle coordination and the presence of urinary incontinence. It is noteworthy that some of their continent participants also showed the presence of PF dysfunction, such as pelvic pain (38%), dyspareunia (14%), and pelvic organ prolapse (6%) [24]. Vesting et al. observed a 20% rate of occurrence of involuntary PFM contraction using vaginal palpation in the supine position in another study of women (*n*=222) 3 months postpartum [18], which was comparable with our occurrence rate (15%). Both our study and Vesting et al. included younger women, a mean age of 26.3 and 33.1 respectively [18]. Antônio et al. in a study of women (*n*=97) with SUI reported the lowest rate of occurrence of involuntary PFM contraction (5%) as assessed by vaginal palpation during a cough in the supine position [22]. Our study found a low occurrence rate among healthy nulliparous women, whereas the aforementioned studies found variable rates among women with PF dysfunction or postpartum.

Three studies reported on the reliability of visual observation, whereas four studies reported on the reliability of vaginal palpation. Vesting et al. calculated slight inter-rater reliability (κ = 0.10) for visual observation, and moderate reliability (κ = 0.51) for vaginal palpation [18]. Slieker-Ten Hove et al. found fair inter-rater reliability (κ = 0.33) for both visual observation and vaginal palpation, and moderate intra-rater reliability for visual observation (κ = 0.54) and vaginal palpation (κ = 0.66) when assessing patients in the supine position during a cough [21]. Vieira et al. reported moderate inter-rater reliability (κ = 0.71) and intra-rater reliability (κ = 0.77) using bi-digital vaginal palpation [24].

As discussed previously, the Devreese study was excluded [19]. The significant variability in inter-rater and intra-rater Kappa values among the studies suggests potential concerns regarding the reliability of our assessment methods in measuring the same phenomenon. This variability could also be attributed to participant heterogeneity, making measurements more challenging owing to factors such as age and the presence of other pelvic floor dysfunctions.

Given the wide range of occurrence rates, the variability in included populations, and the diversity of PF dysfunctions in the previously mentioned studies, it can be hypothesized that PFM contractions may function as an involuntary compensatory mechanism during heightened IAP, potentially leading to a higher prevalence in individuals with PF dysfunctions. Although PFM training may be effective for those experiencing PF dysfunction, the assumption that healthy women normally produce an involuntary PFM contraction in response to IAP needs further research. Finally, none of the studies, except ours, evaluated a population of healthy, nulliparous women. Therefore, our novel findings add to the existing literature and underscore the need for more research.

### Differences in Position

To our knowledge, no studies have investigated the occurrence of involuntary PFM contraction while differentiating between positions. For the visual observations, our study found a significant difference in the PFM contraction occurrence rates between the supine (15%) and standing (5%) positions, which could be attributed to the influence of gravity on the abdominal structures [25]. As such, increased IAP (during the cough) and the subsequent increase in downward pressure could have diminished any noticeable upward movement of the perineum during an involuntary PFM contraction. The standing position also presents a challenge. Even with the assistance of a mirror to enhance the visibility of the perineum, doubts persisted about the feasibility of making precise observations. For vaginal palpation, our study found no significant differences in the rates of occurrence of involuntary PFM contraction between the supine and standing positions.

### Comparative Analysis of Study Findings with the Terminology Reports

In comparing our findings with different IUGA/ICS terminology reports [1–4], we found a high level of agreement with the definition of a PFM contraction as assessed by vaginal palpation, but only partial agreement for visual observation.

### Visual Observation Assessment

The results for the visual observation showed partial concordance with the four terminology reports. Adhering to the terminology reports of Messelink et al. and Haylen et al. [1, 2], we defined the “presence” of an involuntary PFM contraction as either no perineal downward movement or an inward movement due to the guarding action of the PFMs; and its “absence” as a perineal downward movement. We identified an involuntary PFM contraction during a cough in 15% of participants in the supine and in 5% in the standing position. Bø et al. described an involuntary PFM contraction using visual observation as a constriction and inward (ventro-cephalic) movement of the pelvic opening, noting that some participants may also demonstrate controlled or limited downward movement [3]. Our study findings align but vary markedly in degree. Whereas Bø et al. reported a downward movement in some participants, we observed a downward movement in the majority (85–95%) of participants. Using visual observation, Frawley et al. differentiated between a conscious (voluntary) control (i.e., the Knack maneuver) associated with an inward movement of the perineum and a reflexive unconscious response (involuntary) associated with no perineal or downward movement [4]. Our findings on perineal movement align with those of Bø et al. and Frawley et al., both of whom describe perineal movement as occurring in a downward and inward direction. However, our study results contradict Frawley et al.’s definition of the underlying mechanisms (motor control or reflexive response) behind an involuntary PFM contraction, as we observed perineal inward movement in 15% of our study population, despite them receiving no instruction to precontract their PFMs and having no history of PFM training (Table 2).

**Table 2:** Comparison of an involuntary pelvic floor muscle (*PFM*) contraction, by assessment method (visual observation, vaginal palpation) and terminology report

Reference	Mechanism	Visual observation	Vaginal palpation
Messelink et al. [1]	Guarding action of the PFMs	Inward movement, no downward movement	Present
Haylen et al. [2]	Guarding action of the PFMs	Inward movement, no downward movement	Present
Bø et al., 2016 [3]	Involuntary contraction of the PFMs	May demonstrate constriction or controlled, limited downward dorsal perineal movement in response to increased IAP	For palpation it refers to the Messelink Report
Frawley et al., [4]	Rapid increase in IAP: conscious control—coordination (motor control); reflex	Elevation, no change, or (small) degree of perineal descent	Present, reflex contraction not described

### Vaginal Palpation Assessment

Our vaginal palpation definitions and findings aligned with the four terminology reports, whereby an involuntary PFM contraction is identified either by its absence or by its presence. Frawley et al. described the involuntary response as an aspect of coordination and a motor-controlled response to a rise in IAP. It is noteworthy that neither provide output findings for involuntary PFM contractions that occur reflexively during a cough. As the women in our study were not instructed to pre-contract their PFMs during the cough, in line with Frawley et al., we assume that the results are attributable to a reflexive (involuntary) response (Table 2) [4].

Unexpectedly, our study revealed low rates of involuntary PFM contraction among young, healthy women with no PF dysfunction. This raises multiple questions: does an involuntary PFM contraction really represent a “normal” response in healthy women, or did the assessment methods (visual observation, vaginal palpation) fail to accurately measure their intended parameters?

Addressing these questions is especially challenging; hence, the limited research in this area. The literature indicates that PF response involves a complex interaction between involuntary (reflexes, feed-forward movement) and voluntary (motor control, Knack) control, for which our study highlights the need for further exploration. The question of whether visual observation and vaginal palpation methods can discern between involuntary and voluntary PFM contractions also requires further research, as suggested by our inter-rater and intra-rater reliability discussion. Our comparative analysis suggests that the validity of these assessment methods might be influenced by other factors, such as diversity of included populations: participants’ age, parity, the presence/absence of PF dysfunction, and the subjective nature of both methods. Other factors that could impact outcomes, such as the influence of gravity, and types of IAP, might also help to explain the variability in occurrence rates and reliability testing. As evidenced by the limited available literature, it is challenging to research such complex factors.

### Strengths and Limitations

The strength of this study lies in its inclusion of a homogeneous population of young, healthy nulliparous women with no PF dysfunction and their ability to actively contract their PFMs. Further, all assessments were conducted by PF physiotherapists (JdJ, BJ) with extensive experience in evaluating PFMs and knowledgeable with regard to the IUGA/ICS terminology reports. In terms of limitations, it is important to note that both assessment methods, vaginal palpation and visual observation, are subjective: reliant on the evaluator’s observations or perceptions. Moreover, the limited evidence in the literature rendered study-outcome comparisons difficult. Finally, definitions and outcome measures in the terminology reports are, at times, inconsistent and require further clarification.

### Clinical and Research Relevance

The attribution of “voluntary” or “involuntary” to a PFM contraction is theoretical and subjective. Researchers and clinicians generally assume that a PFM contraction is “involuntary” if a woman was not specifically asked to tighten her PFMs or had prior PF training (e.g., the Knack). Our study highlights the challenges in assessing involuntary PFM contractions and the divergence in results based on the use of different assessment methods. More significantly, the low occurrence rates in healthy, nulliparous women raises a key question: is the presence of an involuntary PFM contraction truly indicative of “normal” PF functioning. Our study findings and the comparative analysis with previous studies also raises questions about the accuracy and validity of the visual observation and vaginal palpation assessment methods as a valid measure of involuntary PFM contractions.

Ultimately, as our findings and comparative analysis with previous studies highlight, the reasons for the presence or absence of an involuntary PFM contraction is still poorly understood and necessitates more research:

1. To investigate what the absence of an involuntary PFM contraction really indicates
2. To clarify the underlying mechanisms of an involuntary PFM contraction
3. To develop clear, consistent, consensus-based involuntary PFM contraction definitions, measure and assessment methods
4. To standardize these protocols across terminology reports

## Conclusions

Our study presents novel findings that appear to challenge the existing evidence as well as some definitions (and related outcome measures) in the IUGA/ICS terminology reports. Because the majority of research focused on “voluntary” contractions, and PFM training has been shown to improve symptoms of PF dysfunction (e.g., SUI, POP), the inverse, an “involuntary” PFM contraction, is de facto considered to be part of a normal PF response in healthy women. However, until now, no study had to our knowledge actually assessed involuntary PFM contractions in a cohort of healthy women. In line with the literature, we expected to find high rates of occurrence of involuntary PFM contractions. Conversely, we observed unexpectedly low occurrence rates in our population of young, healthy nulliparous women with no PF dysfunction, as assessed by visual observation and vaginal palpation in both standing and supine positions. This suggests that both assessment methods could lack validity as a method of assessing PFM contractions, but also raises questions regarding the very nature of an involuntary PFM contraction (as defined in the literature) and the role that it actually plays in normal and abnormal PF function. For example, the differences in occurrence rates across various populations and conditions, as documented in both the literature and our study, indicate that the increased presence of involuntary PFM contractions could actually be a compensatory mechanism in response to PF dysfunction, a hypothesis meriting further investigation. Our study underscores the need for more research into defining normal PF functions, a review of our understanding of involuntary PFM contractions, and better standardized guidelines for the assessment methods used to identify and quantify involuntary PFM contractions.
